# Epidemiology, economic, and humanistic burden of hereditary angioedema: a systematic review

**DOI:** 10.1186/s13023-024-03265-z

**Published:** 2024-07-08

**Authors:** Xin Guan, Yanan Sheng, Shuang Liu, Miao He, Tianxiang Chen, Yuxiang Zhi

**Affiliations:** 1 School of International Pharmaceutical Business, China Pharmaceutical University, Nanjing, 211198 China; 2 Medical Affairs, Takeda (China) International Trading Company, Beijing, 100006 China; 3 Department of Allergy, Peking Union Medical College Hospital, Chinese Academy of Medical Sciences & Peking Union Medical College, Beijing, 100730 China

**Keywords:** Hereditary angioedema, Autosomal disorder, Economic cost, Clinical burden, Quality of life

## Abstract

**Background:**

This systematic study aims to assess the global epidemiologic, economic, and humanistic burden of illness associated with all types of hereditary angioedema.

**Methods:**

A systematic search for articles reporting the epidemiologic, economic, and humanistic burden associated with patients with HAE was conducted using English and Chinese literature databases from the inception to May 23, 2022. The selected studies were assessed for their quality and risk of bias. The study was conducted in accordance with Preferred Reporting Items for Systematic Reviews and Meta-Analyses and registered with the International Prospective Register of Systematic Reviews (PROSPERO; CRD42022352377).

**Results:**

In total, 65 articles that met the search inclusion criteria reported 10,310 patients with HAE, of whom 5861 were female patients. Altogether, 4312 patients (81%) and 479 patients (9%) had type 1 and type 2 HAE, respectively, whereas 422 patients (8%) had HAE-normal C1-INH. The overall prevalence of all types of HAE was between 0.13 and 1.6 cases per 100,000. The mean or median delay from the first onset of a symptom of HAE to confirmed diagnosis ranged from 3.9 to 26 years. The estimated risk of death from asphyxiation was 8.6% for patients with HAE. Hospitalization, medication, unnecessary surgeries, doctor visits, specialist services, and nursing costs are direct expenses that contribute to the growing economic burden. The indirect cost accounted mostly due to missing work ($3402/year) and loss of productivity ($5750/year). Furthermore, impairment of QoL as reported by patient-reported outcomes was observed. QoL measures identified depression, anxiety, and stress to be the most common symptoms for adult patients and children.

**Conclusion:**

This study highlights the importance of early diagnosis and the need for improving awareness among health care professionals to reduce the burden of HAE on patients and society.

**Supplementary Information:**

The online version contains supplementary material available at 10.1186/s13023-024-03265-z.

## Introduction

Hereditary angioedema (HAE) is a rare, debilitating, life-threatening genetic disorder characterized by recurrent attacks of subcutaneous and/or submucosal angioedema [1]. Several forms of HAE have been defined based on gene mutations: (1) type 1 HAE identified as C1 inhibitor (C1-INH) deficiency with low levels of C1-INH; (2) type 2 HAE identified as C1-INH dysfunction with normal or slightly increased levels of C1-INH but low functional levels, both type 1 and type 2 are due to mutations of the serine protease inhibitor gene 1 (*SERPING1*); and (3) HAE with normal C1-INH levels (HAE-nC1-INH) including (a) mutations of FXII gene (HAE-FXII), (b) HAE with a mutation in the angiopoietin-1 gene (HAE-ANGPT1), (c) HAE with a mutation in the plasminogen gene (HAE-PLG), (d) HAE with a mutation in the kininogen 1 gene (HAE-KNG1), (e) HAE with a mutation in the myoferlin gene (HAE-MYOF), and (f) HAE with a mutation in the heparan sulfate 3-O-sulfotransferase 6 gene (HAE-HS3ST6); some patients have HAE due to unknown mutations identified as HAE-UNK [2]. C1-INH is an inhibitor of plasma kallikrein and factor XII that are responsible for the generation of bradykinin. An increase in the levels of bradykinin causes extravasation of plasma, which leads to painful swelling. In cases of larynx angioedema, it could be life-threatening to the patients [2].

Hereditary angioedema accounts for approximately 2% of clinical angioedema cases [3]. Although the global prevalence of HAE is estimated at 1:50,000, the true prevalence of HAE remains unclear because the disease is rare [4]. Furthermore, according to the epidemiologic reports, the prevalence of type 1 HAE is observed in the majority of patients (80%-85%), whereas type 2 HAE is present in 15% to 20% of patients [5–8]. HAE-nC1-INH is only accounted for by a minor proportion of patients [9]. An earlier study has observed no major gender or ethnic differences in the HAE type 1/2 [4–7, 10]. However, an analysis reported that HAE-nC1-INH is exclusive to women and postulated it to be related to X-linked dominant mode of inheritance [11]. Likewise, HAE-FXII and HAE-unknown were more pronounced in females with a male to female ration of 1:68 and 1:6.3, respectively [12]. The onset of HAE symptoms varies by age and can occur in children aged < 1 year, with the development of laryngeal attacks occurring usually after the age of 3 years with an increased frequency observed after puberty [13].

Patients with HAE had angioedema attacks including pain and swelling at the extremities, abdomen, genitourinary tract, face, or oropharynx and any other possible site. More often, because of the overlap of clinical symptoms between various forms of angioedema or with other systemic diseases, and the relatively rare of it, HAE remains underreported or misdiagnosed. Consequently, there is a considerable delay in the accurate diagnosis of HAE from the onset of symptoms [1]. This may lead to unnecessary treatments and surgeries further delaying the timely treatment of HAE, which may contribute to a substantial burden in patients with HAE.

HAE attacks are usually variable as well as unpredictable and might be induced by various stimuli. The empirical triggering factors include stress, physical exertion, trauma, infection, hormonal changes, medical interventions, seasonal changes, and the use of certain medicinal products [14]. On average, the frequency of attacks ranges from 1 to 26 per year [10, 15]. But in rare cases, patients have reported 100 attacks per year, which may last up to 5 days [16]. The unpredictability of angioedema attacks, high risk of asphyxia, and the need for emergency intervention often result in a significant burden for patients with C1-INH-HAE[17]. Moreover, the above factors adversely affect the patients’ health-related quality of life (HRQoL) and increase the economic burden.

Many efforts have been taken to quantify the epidemiologic, economic, and humanistic burden of this disease, but the poor comparability between the studies has limited the detection of common issues and real differences. To address this gap, we sought to systematically synthesize the evidence on the epidemiologic, economic, and humanistic burden associated with HAE.

## Methods

This systematic review was conducted in accordance with the Preferred Reporting Items for Systematic Reviews and Meta-Analyses (PRISMA) guidelines and registered with the International Prospective Register of Systematic Reviews (PROSPERO; CRD42022352377).

### Search Strategy

We searched English and Chinese databases for articles related to the epidemiologic, humanistic, and economic burden associated with HAE published from the inception of respective databases until May 23, 2022. The following search terms were used for conducting literature searches: “hereditary angioedema,” “HAE,” “epidemiology,” “prevalence,” “incidence,” “mortality,” “death rate,” “fatality,” “burden of disease,” “healthcare resource utilization,” “cost of illness,” “cost,” “productivity,” “economic,” “economic burden,” “healthcare costs,” “hospitalization,” “direct cost,” “indirect cost,” “quality of life,” “Health-Related Quality Of Life,” “Life Quality,” “activities of daily living,” “patient satisfaction,” “caregiver burden,” “impact of burden,” and “quality adjusted life year.” The search strategies for each database and review process are detailed in the Supplementary file.

### Inclusion and exclusion criteria

We included (1) studies with patients suffering from HAE; (2) studies with the following outcomes (a) epidemiology (prevalence, incidence, mortality rates, and diagnosis delay), (b) economic burden (health resource utilization, direct and indirect cost, inpatient and outpatient visit expenses, family care cost, hospitalization cost, and financial burden cost), or (c) humanistic burden (HRQoL measurements with different tools, disability-adjusted life year [DALY], activities of daily living [ADL], quality-adjusted life-year [QALY], patient satisfaction, and caregiver burden); (3) observational studies (prospective and retrospective cohort studies, cross-sectional studies, and case–control studies) and experimental studies (randomized controlled trials [RCTs], single-arm or nonrandomized controlled trials, and cluster trials); and (4) studies that have been published in English or Chinese databases from the inception to May 23, 2022. Studies that reported costs or cost-effectiveness associated with specific treatments of HAE and consisted of study designs, comments, study protocol, editorials, review articles, case reports, and case series were excluded.

### Study selection

The preliminary screening was conducted based on the title and abstracts according to the predefined eligibility criteria. The full texts of the included articles were further reviewed and examined for relevant outcome as aligned with the eligibility criteria. A full-text screening was conducted independently by 2 researchers, and any disagreements between the reviewers were resolved by discussing with the third independent reviewer.

### Data extraction and quality assessment

Information from the included articles was extracted into a standardized MS Office Excel table. Data related to the author, year of publication, title, study design, demographics of the study population, and outcomes of interest were extracted by 2 independent reviewers with the quality check performed by the third reviewer. Although statistical analysis was not planned, the results were narratively synthesized to identify the common themes and gaps in the evidence. The methodological quality of eligible nonrandomized studies was determined using the Newcastle–Ottawa scale (NOS). The NOS consists of 3 quality parameters with a total of 9 points. Studies with an NOS score of > 6 were considered high-quality studies [18].

## Results

### Study selection

**Fig. 1:**
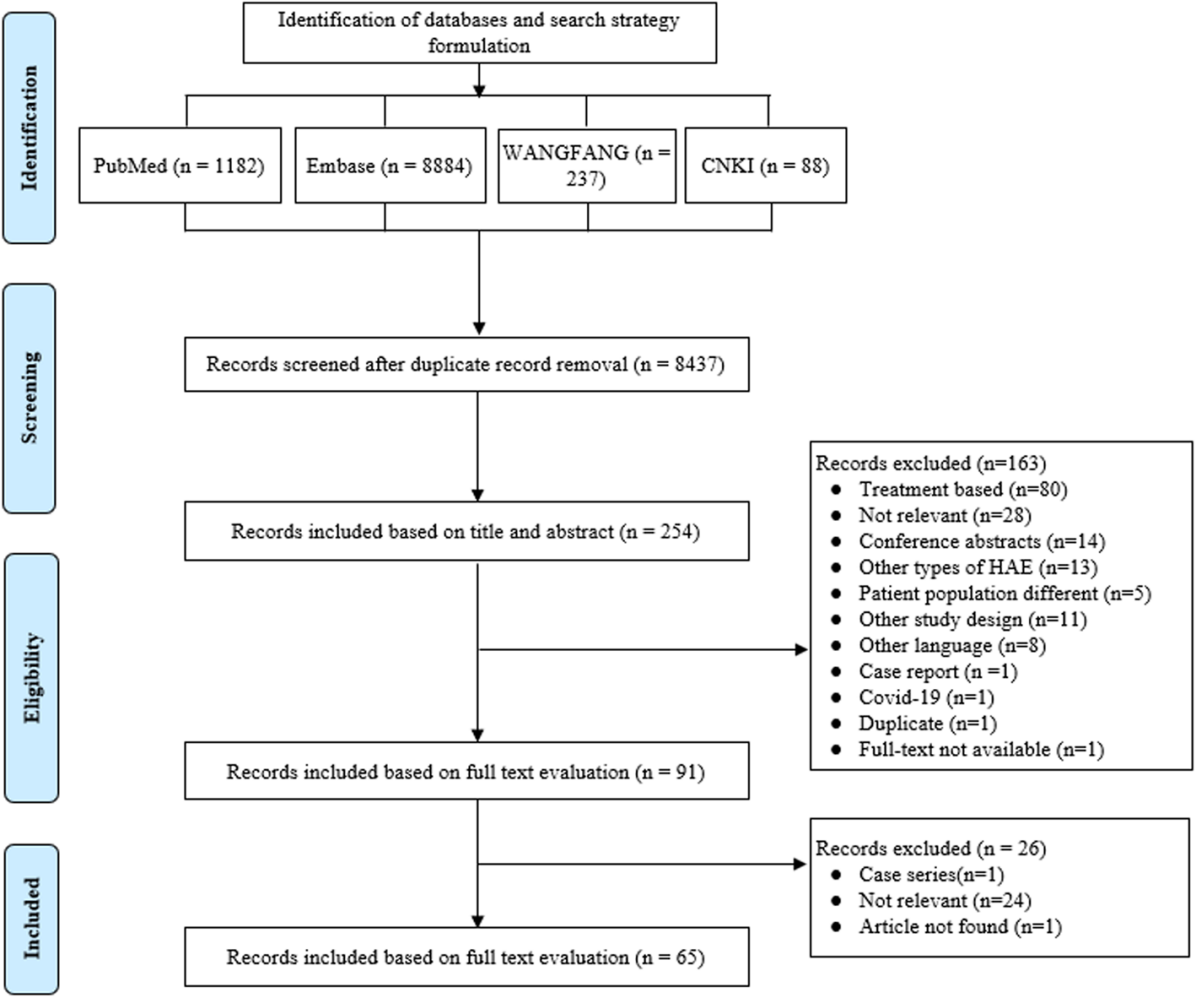
PRISMA flowchart for systematic review. HAE, hereditary angioedema

A total of 10,391 articles were identified from the database search. Titles and abstracts of 8437 articles were screened after eliminating duplicate articles. A total of 254 articles were identified for full-text evaluation based on the abstract review. Finally, 65 full-text articles that were assessed to fulfill the study outcomes were included for the evidence synthesis and quality assessment (Fig. 1). The burden of all types of HAE with respect to epidemiology was reported in 39 articles [7, 18–55], whereas the economic burden and humanistic burden of the disease were reported in 16 [51, 52, 56–68], and 23 articles, respectively [17, 50–55, 57–61, 69–79].

### Clinical characteristics of included studies

The included studies were published from 1997 to 2022, and the study duration ranged from 43 days to 30 years. The studies comprised 7 multinational studies, and the remaining studies included data from 25 countries/regions: the United States (US; *n* = 11); Brazil (*n* = 6); Mainland China (*n* = 6); Germany (*n* = 3); Canada (*n* = 3); Denmark (*n* = 3); Japan (*n* = 3); Turkey (*n* = 3); Sweden, France, and Hungary (*n* = 2 each); and Australia, Belarus, Greece, India, Iran, Italy, New Zealand, Portugal, Puerto Rico, South Africa, South Korea, Switzerland, Taiwan, and the United Kingdom (*n* = 1 each). The included studies were mostly cross-sectional studies (*n* = 37) [54, 55], retrospective observational studies (*n* = 23), and others (*n* = 5). A total of 10,310 patients were evaluated, of whom 5861 were female patients and 3261 were male patients. In total, 4312 patients (81%) had type 1 HAE and 479 patients (9%) had type 2 HAE, whereas HAE-nC1-INH was reported in 422 patients (8%) and the type of angioedema was not identifiable in 122 patients (2%). The key characteristics of eligible studies and quality assessments are provided in Table 1.

**Table 1:** Key characteristics of included studies

Author name	Year	Country/Region	Study design	Population	Study duration	Numbers	Female (n (%))	HAE types^a^	Prevalence	Diagnosis Delay (mean (SD))	Death^b^	Quality assessment
Fragnan NTML et al.	2018	Brazil	Retrospective	Children; adult	December 2009—November 2017	51	38 (74.5)	49;2;0	NR	median(range): 13 (0.25–50)	NR	5
Cui Q et al.	2022	China	Retrospective	Adult	January 2019—July 2020	2	1(50.0)	2;0;0	C4 decreased cohort: 2.43/10,000	25(NA)	NR	3
Jones DH et al.	2022	US	Retrospective	Adult	NR	23	20(87.0)	0;0;23	NR	> 10(NA)	NR	3
Guryanova I et al.	2021	Belarus	Cross-sectional	Children; adult	2010	64	39(60.9)	54;10;0	1/148,000	Median (IQ_25_-IQ_75_): 19.3 (2.4)	NR	5
Ayteki̇n G et al.	2021	Turkey	Retrospective	NR	5-year	38	25 (65.8)	18;20;0	NR	8.84(8.97)	NR	5
Veronez CL et al.	2021	Brazil	Retrospective	Children; adult	March 2012-March 2020	425	316(73.3)	116;9;300	NR	HAE-C1-INH: 21 (15) HAE-F12: 15 (13) HAE-U: 14 (14)	NR	4
Araújo-Simões J et al.	2021	Brazil	Retrospective	Children	First clinical evaluation until December 2018	95	44(46.3)	NR	NR	3.9(NA)	NR	4
Jindal AK et al.	2021	India	Retrospective	Children	January 1996—December 2019	32	11(34.4)	32;0;0	NR	Median (range): 6.5 (0–28)	1	4
Cao Y et al.	2021	China	Cross-sectional	Children; adult	NR	107	58 (54.2)	103;4;0	NR	14.2 (range,0–50)	NR	3
Ohsawa I et al.	2021	Japan	Cross-sectional	Children; adult	June 2019—May 2020	68	39 (67.2)	NR; NR;10	NR	18.8 (range,0–60)	NR	3
Cao Y et al.	2020	China	Cross-sectional	Children; adult	NR	103	56(54.4)	103;0;0	NR	Median (IQR): 11 (6–19.5)	NR	3
Magerl M et al.	2020	Germany	Cross-sectional	Adult	July 2017—April 2018	81	60 (74.1)	NR	NR	18.1 (14.6)	NR	3
Alonso MLO et al.	2019	Brazil	Cross-sectional	Children; adult	NR	107	72 (67.3)	105;2;0	NR	17.7(12.6)	NR	4
Schöffl C et al.	2019	Austria	Cross-sectional	NR	NR	137	77(56.2)	77;19;0;41	1/64,396	15.0 (9.9)	NR	5
Liu S et al.	2019	China	Cross-sectional	Children; adult	NR	96	53 (55.2)	92;4;0	NR	Median (IQR):11.04(6.06 –18.27)	NR	5
Jung JW et al.	2018	South Korea	Retrospective	NR	First diagnosed until 2016	65	44 (67.7)	59;6;0	1.3/1,000,000	7.75(10.54)	NR	3
Coovadia KM et al.	2018	South Africa	Retrospective	Adult	2010–2015	43	28(65.1)	43;0;0	1/140,000	NR	2	5
Zanichelli A	2016	Austria; Brazil; Denmark; France; Germany; Greece; Israel; Italy; Spain; Sweden; United Kingdom	Retrospective	Children; adult	July 2009—January 2016	418	243(58.1)	387;31;0	NR	≥ 1 misdiagnoses:15.0 (13.4); without misdiagnosis:7.0 (13.2)	NR	3
Deroux A et al.	2016	France	Retrospective	Children; adult	Since 2006	57	46(80.7)	0;0;57	NR	12.7(NA)	NR	3
Kargarsharif F et al.	2015	Iran	Cross-sectional	Children; adult	NR	51	26(51.0)	33;18;0	NR	11.02 (11.60)	2	5
Nanda MK et al.	2015	US	Retrospective	Children	10 years	21	6 (28.6%)	NR	NR	Without a family history: median 6.0 With family history: median -0.9	NR	4
Kim SJ et al.	2014	US	Retrospective	Children; adult	1999—2010	600	NR	NR	NR	NR	0.17 (95% CI 0.15–0.18) per million persons per year	7
Psarros F et al.	2014	Greece	Cross-sectional	Children; adult	July 2010—June 2013	116	55(47.4)	NR	NR	16.5(NA)	NR	5
Caballero T et al.	2014	Spain; Germany; Denmark	Cross-sectional	NR	May-December 2011	186	112 (60.2)	NR	NR	12(15)	NR	5
Bork K et al.	2012	Germany	Partly retrospective and Partly prospective	NR	NR	728	388(53.3)	682;46;0	NR	NR	70	3
Kesim B et al.	2011	Turkey	Retrospective	NR	NR	70	42 (60.0)	67;3;0	NR	26.0 (14.4)	NR	4
Bygum A et al.	2009	Denmark	Cross-sectional	Children; adult	2001–2002; 2007–2008	82	42(51.2)	77;5;0	1.41/100,000	16.3 (range, 0–63)	NR	5
Bork K et al	2000	Germany	Retrospective	Children; adult	NR	153	NR	146;7;0	NR	NR	24	3
Winnewisser J et al.	1997	Switzerland	Cross-sectional	NR	NR	59	NR	NR	NR	NR	4	3
Sylvestre S et al.	2021	US	Cross-sectional	NR	July 2021	2122	1463(68.9)	NR	Overall:0.99/100,000 Black patients: 1.64/100,000 White patients:1.47/100,000 Hispanic patients:0.80/100,000	NR	NR	5
Xu YY et al.	2013	China	Retrospective	NR	1982—2011	158	80(50.6)	156;2;0	NR	12.64(NA)	18	6
Wei-Te Lei et al.	2011	Taiwan	Retrospective	NR	2003—2011	19	8 (42.10)	19;0;0	NR	8.45(11.04)	1	4
Balla Z et al.	2021	Hungary	Retrospective	NR	1990–2020	197	109(55.3)	184;13;0	NR	NR	2	7
Banerji A et al.	2020	US	Cross-sectional	Adult	March 17; 2017—April 28; 2017	445	348 (78.2)	349;96;0	NR	8.4 (10.6)	NR	5
Zilberberg MD et al.	2011	US	Retrospective	Children; adult	2006—2007	NA	NR	NR	NR	NR	NR	3
Zilberberg MD et al.	2011	US	Retrospective	Children; adult	2004—2007	NA	NR	NR	NR	NR	All-HAE:145 HAE-PD:9	3
Zilberberg MD et al.	2010	US	Retrospective	NR	2007	NA	NR	NR	NR	NR	NR	3
Wilson DA et al.	2010	US	Cross-sectional	Adult	November 2007—January 2009	457	345(75.5)	NR	NR	NR	NR	3
Javaud N et al.	2019	France	Cluster randomized trial	Adult	March 2013-June 2014	200	74 (37.0)	164;14;22	NR	NR	NR	5
Mendivil J et al.	2021	France; United Kingdom; Spain; Canada; Australia; Switzerland; Germany; Austria	Cross-sectional	Adult	July–October 2018	242	163 (67.4)	198;44;0	NR	9.3 (11.0)	NR	4
Hews-Girard J et al.	2021	Canada	Cross-sectional	Adult	NR	17	13 (76.5)	11;6;0	NR	NR	NR	5
Nunes FL et al.	2021	Brazil	Prospective trial	Children; adult	14 months	33	18 (54.5)	33;0;0	NR	NR	NR	6
Forjaz MJ et al.	2021	Spain; Hungary; Austria; Germany; Argentina; Brazil; Canada; Denmark; Israel; Poland; Romania	Prospective observational	Adult	NR	290	200(69.0)	232;58;0	NR	NR	NR	5
Ohsawa I et al.	2015	Japan	Cross-sectional	Children; adult	March—May 2014	171	117 (68.4)	99;9;3;60	NR	13.8 (range, 0–58)	NR	5
Aygören-Pürsün E et al.	2014	Spain; Germany; Denmark	Cross-sectional	Children; adult	May-December 2011	164	100 (61.0)	NR	NR	12(NA)	NR	3
Jolles S et al.	2014	UK	Retrospective	Children; adult	2010–2012	376	NR	320;23;4	NR	type 1: 10(NA) type 2: 18(NA)	NR	5
Lumry WR et al.	2010	US	Cross-sectional	Adult	November 2007—January 2008	457	345 (75.5)	NR	NR	NR	NR	3
Lindsay K et al.	2021	New Zealand	Retrospective	Adult	1st June 2015-31st December 2019	38	20 (52.6)	29;9;0	NR	Without a family history: 13.2(NA)	NR	4
Iwamoto K et al.	2021	Japan	Cross-sectional	Children; adult	2016—2017	70	55(78.6)	NR	NR	15.6(13.3)	NR	4
Savarese L et al.	2021	Italy	Cross-sectional	Adult	NR	28	20 (71.4)	NR	NR	NR	NR	4
Balla Z et al.	2021	Hungary	Prospective trial	Adult	2016—2018	125	72(57.6)	NR	NR	NR	NR	6
Lee EY et al.	2021	Canada	Cross-sectional	Adult	NR	72	53 (73.6)	NR	NR	NR	NR	3
Kuman Tunçel Ö et al.	2019	Turkey	Cross-sectional	Adult	NR	33	19 (57.6)	30;3	NR	19.8(9.4)	NR	5
Liu S et al.	2019	China	Cross-sectional	Adult	NR	104	57 (54.8)	101;3;0	NR	NR	NR	5
Arce-Ayala YM et al.	2019	Puerto Rico	Cross-sectional	Children; adult	November 2015-April 2016	32	25 (83.3)	13;4;3;11	NR	NR	NR	3
Kessel A et al.	2017	Hungary; Israel	Cross-sectional	Children	NR	33	19(57.6)	NR	NR	NR	NR	4
Aabom A et al.	2017	Denmark	Cross-sectional	Children	May 2013—August 2014	14	6(42.9)	13;1;0	NR	NR	NR	2
Nordenfelt P et al.	2017	Sweden	Cross-sectional	Adult	May—October 2016	64	38(59.4)	60;4;0	1.61/100,000	NR	NR	5
Engel-Yeger B et al.	2017	Israel; Hungary	Cross-sectional	Children	NR	34	19 (55.8)	34;0;0	NR	NR	NR	4
Jindal NL et al.	2017	Canada	Cross-sectional	Adult	NR	21	20 (95.2)	NR	NR	NR	NR	5
Nordenfelt P et al.	2014	Sweden	Cross-sectional	Children; adult	NR	103	54(52.4)	NR	1/66,000	NR	NR	4
Gomide MACMS et al.	2013	Brazil	Cross-sectional	Children; adult	NR	35	25(71.4)	NR	NR	NR	NR	2
Luz S et al.	2011	Portugal	Cross-sectional	NR	NR	25	17(68.0)	NR	NR	NR	NR	3
Aabom A et al.	2015	Denmark	Cross-sectional	NR	2009	27	6(22.2)	NR	NR	NR	NR	3
Fouche AS et al.	2014	US	Cross-sectional	Adult	NR	26	12(46.2)	22;4;0	NR	NR	NR	3

*HAE* Hereditary angioedema

^a^HAE type: type1; type2; HAE-nC1-INH; non-identification

^b^Death due to asphyxia

### Epidemiologic burden

#### Diagnosed prevalence of HAE

As reported in 8 studies, the prevalence of HAE ranged between 0.13 and 1.6 cases per 100,000 (Table 1). The prevalence rates were low across all included studies. In Sweden, the estimated prevalence rates for 2011 and 2016 were 1.5 and 1.6 cases per 100,000, respectively [54, 55]. In South Korea, the prevalence was 0.13 cases per 100,000 [33], whereas in Denmark and Austria, the estimated prevalence was 1.4 and 1.6 cases per 100,000, respectively [31, 44]. Similarly, the estimated prevalence rates in Belarus, South Africa, and the US were between 0.7 and 1.0 cases per 100,000 [21, 34, 47]. A study conducted in the US simultaneously reported the prevalence of HAE among ethnic groups. The prevalence of HAE in Black patients (1.64 cases per 100,000) was almost similar to that of White patients (1.47 cases per 100,000), whereas it was lower among Hispanic patients (0.80 cases per 100,000) [47]. Furthermore, according to a study conducted in Tongji Hospital in China, the prevalence of HAE was reported to be 2.43 cases per 10,000 in patients with decreased complement 4 level [19].

#### Risk of death in patients with HAE

One study reported a low age-adjusted mortality (the ratio of the number of deaths in a specified time to a given population) of 0.17 (95% CI, 0.15–0.18) per million persons per year for HAE in the US [39]. Additional 9 studies reported 124 deaths caused by asphyxiation (due to laryngeal edema) among 1440 patients, which leads to an estimated 8.6% of risk of death from asphyxiation for patients with HAE. One of the 9 studies reported the lifespan of patients with undiagnosed HAE type 1/2 who died of asphyxiation was shorter than that of patients with undiagnosed HAE type 1/2 who died of other causes (40.8 years vs 72.0 years) [42]. Furthermore, a descriptive epidemiologic study conducted in the US evaluated the death among all HAE hospitalizations and HAE-related hospitalizations [66]. The study observed that 145 deaths occurred during all HAE hospitalizations (*n* = 10,125) and 9 deaths occurred during 3216 HAE-related hospitalizations (Table 1).

#### Diagnosis of HAE

The delay in diagnosis was reported by 34 studies with the mean or median range of 3.9 to 26 years from the first onset of HAE symptoms to the confirmed diagnosis (Table 1). In 25 articles, the mean or median delay was reported to be > 10 years, indicating there was a widespread misdiagnosis of HAE globally. One of the studies found that patients without family history had a longer delay than those with family history (6.0 years vs -0.9 years) [38], whereas another study found that the delay in diagnosis was shorter in patients with HAE type 1 than those with HAE type 2 (10 years vs 18 years) [64].

### Economic burden

Direct costs associated with HAE were reported in 6 studies, which assessed the treatment costs and health care utilization (Table 2). The major components accounting for the increase in economic costs were hospitalization, treatments, unnecessary surgeries, doctors’ visits, specialist services, and nurse costs. Three studies conducted in the US estimated the hospital costs to be $17,335 per year and about $4000 for a single hospitalization [56, 65, 67]. The annual medication costs to reduce the number of attacks and to manage the chronic disease were $235 and $2013, respectively [67]. A study by Javaud et al. conducted in France between 2013 and 2014 reported an increase in the medication cost of €10,038 ± €10,334 and €10,287 ± €8260 at 12 and 24 months, respectively, in patients with HAE [59]. The emergency department (ED) cost for a single visit was $1465 as reported by Zilberberg et al., whereas the annual ED cost was $2603 as reported by Wilson et al. [65, 67]. The other procedural cost accounted for $978 to $3510 among the US patients and €135 among French patients with HAE [59, 67]. According to a study by Wilson et al., outpatient clinic cost comprised the least expenditure with an estimated total cost of $189 [67]. Wilson et al. and Javaud et al. reported the total direct costs with annual medical expenditure of $25,884 and €10,296 for patients from the US and France, respectively [59, 67].

**Table 2:** Direct costs related to HAE

Study name	Country /region	Hospitalization cost	Medication cost	Outpatient’s cost	Emergency visit	Other procedures	Total Direct cost
Banerji et al. 2020 [52]	US	NR	NR	NR	NR	$1,000	NR
Zilberberg et al. 2011 [56, 66]	US	NR	NR	NR	HAE-PD: $1,465	NR	NR
Zilberberg et al. 2011 [56, 66]	US	HAE-PD: $4,760	NR	NR	NR	NR	NR
Zilberberg et al. 2010 [65]	US	Around $4,000 for a single hospitalization	NR	NR	NR	NR	NR
Wilson et al. 2010 [67]	US	Hospital stays: $17,335	Acute attacks: $235 Chronic disease management: $2,013 Total: $2,248	Clinic or physician’s office treatment: $189	$2,603	Procedure cost: $978 Routine visit costs: $2,532 Total: $3,510	Total direct medical costs of acute attacks: $21,339 Total direct medical costs for chronic disease management (treatment outside acute attacks): $4,545 Total direct medical costs $25,884 annually
Nicolas Javaud et al. 2019 [59]	France	0-to-12-month follow-up: Cost: €122 ± 176 12-to-24-month follow-up Cost: €118 ± 180 (€ = $1.11 in 2015.)	0-to-12-month follow-up: Drug cost: €10,038 ± 10,334 12- to 24-month follow-up: Drug cost: €10,287 ± 8,260	NR	NR	0-to-12-month follow-up: ED visits + transportation: €99 ± 25 Consultation GP/specialist: €26 ± 10 Nurse: €10 ± 2 12-to-24-month follow-up: ED visits + transportation: €101 ± 24 Consultation GP/specialist: €27 ± 10 Nurse: €10 ± 2	Total average health care cos during the first year: €10,296 ± 17,828 Total average health care cos during the second year: €10,544 ± 17,525

*Abbreviations*: *ED* Emergency department, *GP* General physician, *HAE* Hereditary angioedema, *PD* Principal diagnosis

Twelve studies reported indirect economic burden that included various productivity measures (Table 3). Among them, absenteeism from work was 5.9% to 31.1% because of HAE attacks. The presenteeism among patients who were physically present at work was approximately 20%, ranging from 10% to 24.6%. Furthermore, Wilson et al. reported a loss of $5750 because of decreased productivity at work affecting income. In addition, the study also reported the loss of wages for missed work because of a single attack approximated to be $525. Therefore, missed work because of acute attacks resulted in an income loss of $3402 annually, and for chronic disease management, the income was reduced to almost $6512 because of working fewer hours as compared with working full time [67]. There was variation in the number of days missing from work/school because of different time duration. In general, the average number of missed days in 1 year was between 1.7 and 19.9 days, and in total, the indirect cost was $16,108 annually.

**Table 3:** Indirect economic burden of HAE

Study Name	Absenteeism (mean ± S.D.)	Presenteeism (mean ± S.D.)	Work productivity losses (mean ± S.D.)	Activity impairment (mean ± S.D.)	Loss of income due to productivity loss	Loss of income due to reduced working hours	Number of days lost due to HAE attack	Total indirect cost
Mendivil et al. 2021 [51]	7.87% ± NA	24.59% ± 28.65%	24.18% ± 30.03	33.88% ± 31.20%	NR	NR	NR	NR
Hews-Girard et al. 2021 [57]	31.1% ± NA	10% ± 18%	27% ± NA	20.6% ± 21.1%	NR	NR	NR	NR
Leonel Nunes et al. 2021 [58]	7.29% ± 18.52	19.29% ± 28.41	21.14% ± 32.39%	25.00% ± 26.24%	NR	NR	NR	NR
Banerji et al. 2020 [52]	5.9% ± 14.1%	23.0% ± 25.8%	25.4% ± 28.1%	31.8% ± 29.7%	NR	NR	NR	NR
Ohsawa et al. 2015 [62]	NR	NR	NR	NR	NR	NR	Mean 1.7 absent days per year	
Pürsün et al. 2014 [63]	NR	NR	NR	NR	NR	NR	Days missing from work/school on average per year: 19.9 ± 35.0 day	NR
S. Jolles et al. 2014 [64]	NR	NR	NR	NR	NR	NR	Days lost from work/school or where activities of daily living could not be performed: 9 ± 24 days per year	NR
Lumry et al. 2010 [60]	9.4% ± 19.2%	NR	33.5% ± 25.8%	45% ± 30.2%	NR	NR	Numbers of missed day due to most recent attack: Work days 3.3 ± 14.4 school days 1.9 ± 0.8 leisure days 2.7 ± 3.0	NR
Wilson et al. 2010 [67]	NR	NR	33.5% ± NA	NR	$5,750 per patient per year	Average cost of lost wages for missed work due to a single attack (per patient annually): $525 Annual cost of missed work due to acute attacks: $3,402 Annual reduced income because working less than full time: $6,512	Numbers of missed day due to most recent attack: Overall: 3.3 ± 14.4 days Mild attack: 2.2 ± 3.3 days Moderate attack: 1.8 ± 1.0 days Severe attack: 5.5 ± 22.9 days	Total indirect costs: $16,108
Lindsay et al. 2021 [68]	NR	NR	NR	NR	NR	NR	Mean days off work over one year: 16 days (range 1–104)	NR
Iwamoto et al. 2021 [69]	NR	NR	NR	NR	NR	NR	Days absent from work/school in year: 17.5 ± 4.4 days before diagnosis 10.2 ± 3.6 days after diagnosis	NR

*Abbreviations*: *HAE* Hereditary angioedema, *S.D.* Standard deviation

### Humanistic burden

The quality of life (QoL) was reported in a total of 23 publications. In 9 studies, the most frequently used measure to assess the HRQoL for patient-reported outcome (PRO) was 36-item Short Form health survey (SF-36). Other PRO measures used to assess the HRQoL in patients with HAE included the Angioedema Quality-of-Life Questionnaire (AE-QOL; *n* = 4); Hereditary Angioedema Quality-of-Life Questionnaire (HAE-QoL; *n* = 4); the EuroQol-5 Dimension (EQ-5D; *n* = 3); Pediatric Quality of Life Inventory (Peds-QL; *n* = 3); 12-item Short Form Health Survey (SF-12; *n* = 3); Hospital Anxiety and Depression Scale (HADS; n = 2); Visual Analog Scale (VAS; *n* = 2); Toronto Alexithymia Scale (TAS; *n* = 1); Emotion Regulation Checklist (ERC; *n* = 1); Perceived Stress Scale (PSS; *n* = 1); Depression, Anxiety, Stress Scale-21 (DASS-21; *n* = 1); State-Trait Anxiety Inventory for Children (STAIC; n = 1); Children's Dermatology Life Quality Index (CDLQI; *n* = 1); Research and Development-36 (RAND-36; *n* = 1); Hamilton Depression Inventory-Short Form (HDI-SF; *n* = 1); and Hamilton Depression Rating Scale (HDRS; *n* = 1). Table 4 lists the summary of studies reporting humanistic burden in HAE.

**Table 4:** Humanistic burden in HAE

Study Name	SF-36	AE-QOL	HAE-QoL	EQ-5D	PedsQL	SF-12	Other
Savarese et al. 2021 [17]	NR	NR	NR	NR	NR	NR	TAS:43.3 (12.9) ERC:4.4 (0.8) PSS:18.2 (7)
Mendivil et al. 2021 [51]	NR	47.14 (20.69)	NR	NR	NR	PCS: 49.26 (9.30) MCS: 43.09 (11.23)	HADS:13.43 (8.17)
Hews-Girard and Goodyear 2021 [57]	*P* < 0.001 compared with Canadian normative data	39 (18.2)	NR	NR	NR	NR	DASS-21 depression score:6.8 (10.2) DASS-21 anxiety score: 6.2 (8.2) DASS-21 stress score: 10(10.2)
Balla et al. 2021 [50]	NR	Median (IQR) 20.6 (5.9, 36.8)	NR	NR	NR	NR	NR
Nunes et al. 2021 [58]	NR	NR	Mean (CrI): Δscore:15.2 (1.23–29.77) at 8 months Δscore:26 (14.56–39.02) at 14 months	NR	NR	NR	NR
Lee et al. 2021 [70]	NR	NR	102 (23)	NR	NR	NR	NR
Banerji et al. 2020 [52]	NR	NR	93.1 (24.9)	NR	NR	PCS:48.6 (9.9) MCS: 44.9 (10.9)	HADS Anxiety 4.3(3.5), depression 2.5(2.9) for Attack-free; anxiety 8.8 (4.7), depression 6.4 (4.8) for 13 or more attacks
Kuman Tuncel et al. 2019 [53]	Score of ERF, SF, GH, BP, PRF subscales lower than population norms (*P* < 0.01)	NR	NR	NR	NR	NR	NR
Shuang Liu et al. 2019 [71]	PCS: 49.81(7.08) MCS: 44.76 (9.18)	NR	NR	NR	NR	NR	NR
Javaud et al. 2019 [59]	NR	NR	NR	12 months: 0.71(0.12) 24 months: 0.70(0.13)	NR	NR	NR
Arce-Ayala et al. 2019 [59]	PCS:40.91(NA) MCS: 41.57(NA)	NR	NR	NR	NR	NR	NR
Kessel et al. 2017 [73]	NR	NR	NR	NR	C1-INH-HAE vs controls Hungary: 81.52(14.18) vs 92.48 (5.54) Israel: 79.93 (11.98) vs 87.42 (8.15)	NR	STAIC (HAE vs controls) Anxiety state 44.74(10.56) vs 38.76(10.67) Anxiety trait 29.21(5.16) vs 25.23(4.09)
Aabom et al. 2017 [74]	NR	NR	NR	NR	C1-INH-HAE vs controls Child Self-Report 84.0(18.6) vs 82.9(NA) Parent Proxy-Report 83.4(18.8) vs 81.3(NA)	NR	CDLQI: 2.0(5.9) disease-specific questionnaire:5.6(10.0) VAS line: 86.0(23.3) VAS smiley: 84.1(19.8)
Nordenfelt et al. 2017 [54]	NR	Median (range) 36.8 (0–91.7)	NR	0.84 (-0.02–1.00)	NR	NR	VAS: 80 (25–100) RAND-36: The scores of the nine dimensions: 50–100
Engel-Yeger et al. 2017 [75]	NR	NR	NR	NR	C1-INH-HAE vs controls Hungary: 81.81(13.83) vs 80.22(14.82) Israel: 79.93 (11.98) vs 86.39(5.71)	NR	NR
Jindal et al. 2017 [76]	PCS: 49.1(NA) MCS: 50.4(NA)	NR	NR			NR	NR
Nordenfelt et al. 2014 [55]		NR	NR	Today vs attack 0.825 (0.207) vs 0.512 (0.299)		NR	NR
Gomide et al. 2013 [77]	The scores of the eight dimensions: 51.03 to 75.95	NR	NR	NR	NR	NR	NR
Luz et al. 2011 [78]	The scores of the eight dimensions: 49.16 to 83.20 vs 55.83 to 75.27 (HAE patient vs Reference)	NR	NR	NR	NR	NR	NR
Lumry et al. 2010 [60]	NR	NR	NR	NR	NR	HAE patient vs Reference PCS: 43.7(10.2) vs 49.6(9.9) MCS: 42.6(10.1) vs 49.4(9.8)	HDI-SF 8.1 (6.5) vs 3.1(3.0) (HAE patient vs Reference)
Aabom et al. 2015 [79]	The scores of the eight dimensions: 62.8 to 92.9 vs 58.3 to 87.4 (HAE patient vs Reference)	NR	NR	NR	NR	NR	NR
Fouche et al. 2014 [83]	NR	NR	NR	NR	NR	NR	17-item HDRS: 7.1 (6.2) vs 3.2 (3.2) for HAE cohort and General population cohort 21-item HDRS: 8 (6.5) 29-item HDRS: 11 (8.9)
Forjaz MJ et al. 2021 [61]	PCS: 49.7(8.8) MCS: 46.2(10.4)	NR	95.5(25.5)	NR	NR	NR	NR

AE-QoL, Angioedema Quality of Life Questionnaire, scores were transformed to a linear scale of 0–100(0 = none; 0–25 = mild; 26–75 =moderate and > 75 = severe). HAE-Qol, Hereditary Angioedema Quality of Life Questionnaire, total score ranges from 25 to 135, higher scores represent better HRQoL. CDLQI, Children's Dermatology Life Quality Index, score range 0-30, higher scores reflect more impaired HRQoL. DASS-21, Depression Anxiety Stress Scale-21, Depression (normal 0-4, mild 5-6, moderate 7-10, severe 11-13, extremely severe ≥14); Anxiety (normal 0-3, mild 4-5, moderate 6-7, severe 8-9, extremely severe ≥10); Stress (normal 0-7, mild 8-9, moderate 10-12, severe 13-16, extremely severe ≥17). ERC, Emotion Regulation Checklist, the mean values for both males and females within the normative samples can be referred as cut-off values for the index. HADS, Hospital Anxiety and Depression Scale, anxiety and depression subscale scores range from 0 to 21, 0-7 = Normal, 8-10 = mild, 11-14=moderate, 15: severe psychological morbidity. HDRS, Hamilton Depression Rating Scale, higher values correlate with more severe depressive symptoms. Peds-QL, Pediatric Quality of Life, 0-100-point scale, a higher total score indicates a better HRQoL. PSS, Perceived Stress Scale, a 10-item self-reported scale, measures the degree to which situations in one’s life are appraised as stressful. Stress was assessed as low with a score < 13, moderate with a score of 14–26, and as highly perceived when the score was > 27. RAND-36, Research and Development, average score between 0 (worst) and 100 (best), a higher total score indicates a better HRQoL. SF-36, 36-item Short Form; average score between 0-100, a higher total score indicates a better HRQoL. SF-12, 12-Item Short Form Health Survey, scores range from 0 to 100 with higher score indicating better physical and mental health. STAIC, State-Trait Anxiety Inventory for Children, total scores for state and trait range from 20 – 80, higher scores indicate greater anxiety. TAS, Toronto Alexithymia Scale, a 20-item self-reported questionnaire which evaluates alexithymia, the difficulty in recognizing and naming and describing one’s own emotions. A score of < 51 indicates absence of alexithymia, a score of 52–60 indicates a possible alexithymia, and a score of > 61 indicates alexithymia. EQ-5D, The EuroQol 5 Dimension, range 0-1, higher scores reflect better health. VAS, Visual Analogue Scale, 0-100, higher scores reflect better general health. HDI-SF, the range of score is 0-33, with higher scores indicating more depression symptom. nonvalidated tool, range 0-52, higher scores reflect more impaired HRQoL

*HAE* Hereditary angioedema, *MCS* Mental component summary score, *PCS* Physical component score, *QoL* Quality of life, *ERF* Emotional role functioning, *S*F Social functioning, *GH* General health, *BP* Bodily pain, *PRF* Physical role functioning

The mean overall summary scores for the physical component summary (PCS) in the SF-36 survey ranged from 40.9 to 49.8 and for the mental component summary (MCS) ranged from 41.6 to 50.4. Three studies from Brazil, Portugal, and Denmark reported the mean scores of 8 domains of SF-36 ranging from 51.0 to 76.0, 49.2 to 83.2, and 62.8 to 92.9 [77–79], respectively. The mean scores in at least 1 dimension were significantly lower for the HAE population compared with the normal population in 5 studies [53, 57, 71, 72, 76], whereas another 2 studies reported that patients with HAE had QoL scores similar to the reference population [78, 79]. Three studies reported the scores of the PCS and MCS in the SF-12 survey, ranging from 43.7 to 49.26 and 42.6 to 44.9, respectively [51, 52, 60]. Nordenfelt et al. evaluated the utility of EQ-5D to describe the current health state and the state during their most recent HAE attack, which indicated an impaired HRQoL for patients with HAE both during and between attacks [55]. Another 2 studies that measured the HRQoL using EQ-5D demonstrated an impairment of QoL as well, with a health utility of 0.7 for French patients and of 0.8 for Swedish patients [54, 59]. The scores of VAS were reported in 2 studies with the value of 80.0 for adult patients and 86.0 for pediatric patients [54, 74].

A disease-specific questionnaire such as AE-QoL was also used to assess the impact of angioedema on daily life for 4 weeks before answering the questionnaires. The instrument involved a 17-item questionnaire assessing the impairment of HRQoL from 4 dimensions (functioning, fatigue/mood, fears/shame, and nutrition), and a higher score indicated the severity of impaired HRQoL. The AE-QoL scores in 4 studies varied from 20.6 to 47.1. HAE-QoL, another 25-item disease-specific questionnaire, assessed the extent to which angioedema has affected daily life for the last 6 months from 7 dimensions (treatment difficulties, physical functioning and health, disease-related stigma, emotional role and social functioning, concern about offspring, perceived control over illness, and mental health), with the higher score representing better HRQoL. The mean scores of the included studies ranged from 93.1 to 102. Nunes et al. showed substantial improvement in HAE-QoL scores at 8 and 14 months compared with baseline because of a systematic intervention (Δ score: 15.2 at 8 months; Δ score: 26.0 at 14 months) [58].

Peds-QL is the most frequently used tool to evaluate the HRQoL in pediatric patients with HAE, with a higher total score indicating a better HRQoL. Two of the 3 studies measured using Peds-QL demonstrated a lower HRQoL for pediatric patients with HAE than healthy children [73, 75], whereas the third study observed comparable HRQoL scores for pediatric patients with HAE type 1/2 with normal scores for healthy children [74]. Aabom et al. developed a nonvalidated, disease-specific tool using Peds-QL as a structural model to measure the impact of HAE on pediatric patients, with a score of 5.6 ± 10.0. The CDLQI designed to measure physical discomfort, social discomfort, and activity limitation showed a CDLQI score of 2.0 ± 5.9 for pediatric patients [74].

Depression, anxiety, stress, and alexithymia are the most common symptoms for both adult patients and children as proved by DASS-21, PSS, HDI-SF, HDRS, STAIC, TAS, and ERC (see Table 4). Another uncommon instrument has been used to report the QoL in the studies. The RAND-36 is a generic instrument (similar to SF-36) evaluating the HRQoL from 9 dimensions, with 0 for the worst and 100 for the best. Nordenfelt et al. reported median scores of 9 dimensions of RAND-36 ranging from 50 to 100 [54].

## Discussion

To the best of our knowledge, this systematic review is the first to provide a comprehensive understanding of the epidemiologic, economic, and humanistic burden of HAE. This review indicates that HAE is associated with a substantial burden and will undoubtedly become more pronounced with rising awareness of the disease globally. At present, because of rarity and limited symptom specificity, HAE is indeed often misdiagnosed, leading to a significant delay (> 10 years) in correct diagnosis. Besides, a lack of awareness among health care professionals, limited availability of diagnostic tests, and incorrect treatment restrict the timely and optimal management of HAE [80, 81]. As a result, it has been found that 8.6% of patients with HAE have experienced laryngeal edema, which has led to death caused by asphyxiation. These concerning statistics emphasize the critical need for early diagnosis and increased awareness of the disease.

The HRQoL is the patients’ perception regarding the multidimensional impact of the disease [82]. Evidence from this review illustrates the negative impact of HAE on the QoL. Twenty-three studies assessed the burden of HAE on the QoL of patients; however, only 3 reported QoL of children aged 2 to 18 years with HAE highlighting a significant knowledge gap [73–75]. In this study, we observed that most patients were provided with an SF-12/SF-36 survey questionnaire or an HAE-QOL/AE-QOL questionnaire. The majority of the studies showed poorer PCS and MCS in patients with HAE relative to the control. There was a significant association of psychological implications such as anxiety and depression with HAE identified by either high score values on rating scales or during conversations with the participants [83]. HAE had a significant negative impact on the QoL both during and between attacks that reflected on absenteeism at work or school [55]. The World Allergy Organization guidelines about C1-INH-HAE suggest considering the HRQoL when determining maintenance treatment, and HAE experts advise to assess the HRQoL annually [2, 84]. Data derived from large populations are necessary to accurately measure the HRQoL in patients with C1-INH-HAE, its trigger factors, and the effects of therapeutic interventions.

To our knowledge, only 2 studies from the US and France have estimated the economic burden associated with HAE. Although the evidence is sparse, the present review identified hospitalization cost, medication cost, and other procedural costs such as surgery, physician visits, and nursing services ($1000-$3510 among the US patients and €135 among French patients with HAE) to be the main components of direct costs, whereas outpatient visits and other outpatient services ($189) were the minimal components of economic burden as reported in the included studies [59, 67]. Economic assessments of HAE indicated decreased work productivity because of disease burden added to the indirect costs. The loss of income because of reduced productivity was $5750 per patient per year as reported by Wilson et al. and lost wages for missed work because of a single attack was estimated to be ~ $525; conversely, the lost wages per annum would be ~ $3402; and the reduction in income because of absenteeism was ~ $6512 [67]. These estimates indicate a considerable economic burden associated with HAE ascertaining the need to prevent functional limitation and improving the QoL for patients is vital in reducing absenteeism.

Meanwhile, there was a huge variation in the reported data pertaining to the cost; few studies provided a detailed breakdown of direct and indirect costs, whereas other studies described only major cost categories, which limited the comparisons between studies. Finally, variability in outcome measures was observed across studies. The study heterogeneity in terms of patient characteristics and study setting (eg, recruitment at secondary or tertiary clinics and claims databases) across the included articles may have contributed to the wide ranges of the observed data in the results. Future studies using standardized approaches to conduct and report the burden of illness would reduce this data heterogeneity and enable better burden comparisons between studies. With the approval of new drugs for HAE globally, there is an urgent need to determine the direct medical costs incurred by patients using these drugs.

## Conclusion

The lack of comprehensive epidemiologic data on the incidence of HAE creates a knowledge gap regarding the true overall burden of HAE on society. However, there is considerable evidence indicating that delayed diagnosis of HAE is associated with decreased physical function, increased risk of mortality, negative psychological impact, and higher direct and indirect costs. This review compiles evidence highlighting the need for early diagnosis, improved disease management, and increased awareness among health care professionals to mitigate the excessive burden on patients.

### Supplementary Information

Supplementary Material 1.

## Data Availability

The data included in this report are from the published literature; all articles meeting the search criteria are listed and full publication details are provided.
