# Supplementary material for: Epidemiology, economic, and humanistic burden of hereditary angioedema: a systematic review
Source: Orphanet J Rare Dis. 2024 Jul 8;19:256. doi: 10.1186/s13023-024-03265-z (PMC11229247; doi:10.1186/s13023-024-03265-z)
Supplement: Supplementary file 1 — Supplementary Material 1. [file 13023_2024_3265_MOESM1_ESM.docx]

**SUPPLEMENTARY APPENDIX**

**Epidemiology, Economic and Humanistic Burden of Hereditary Angioedema: A Systematic Review**

**Authors:** Xin Guan^1^, Yanan Sheng^2^, Shuang Liu^3^, Miao He^2^, Tianxiang Chen^2^, Yuxiang Zhi^3*^

**Affiliation**

1. School of International Pharmaceutical Business, China Pharmaceutical University, 211198, Nanjing, China,

2. Medical Affairs, Takeda (China) International Trading Company, 100006, Beijing, China

3. Department of Allergy, Peking Union Medical College Hospital, Chinese Academy of Medical Sciences & Peking Union Medical College, 100730, Beijing, China

***Corresponding author**

Yuxiang Zhi, Department of Allergy, Peking Union Medical College Hospital, Chinese Academy of Medical Sciences & Peking Union Medical College, 100730, Beijing, China, yuxiang_zhi@126.com

**Search string used for literature search:**

The following search string was used for the screening of relevant literature in English and Chinese databases with minor changes in Boolean signs to suit the database:

**PubMed**

(hereditary angioedema) AND ((epidemiology) OR (prevalence) OR (incidence) OR (burden of disease) OR (disease burden) OR (burden of illness) OR (occurrence) OR (diagnosis) OR (global burden of disease burden) OR (mortality) OR (death rate) OR (mortality rate) OR (death) OR (survival) OR (fatality) OR (Laryngeal Edema) OR (Asphyxia) OR (Suffocation) OR (Airway Obstruction) OR (healthcare resource utilization) OR (cost of illness) OR (cost) OR (costs) OR (productivity) OR (economic) OR (economic burden) OR (healthcare costs) OR (hospitalization) OR (hospitalisation) OR (health resource utilization) OR (utilization) OR (healthcare utilization) OR (health service utilization) OR (direct cost) OR (indirect cost) OR (quality of life) OR (qol) OR (HrQoL) OR (Health-Related Quality Of Life) OR (Health Related Quality Of Life) OR (Life Quality) OR (activities of daily living) OR (patient satisfaction) OR (caregiver burden) OR (impact of burden) OR (quality adjusted life year))

**EMBASE**

('hereditary angioedema'/exp OR 'hereditary angioedema' OR (hereditary AND ('angioedema'/exp OR angioedema))) AND ('epidemiology'/exp OR epidemiology OR 'prevalence'/exp OR prevalence OR 'incidence'/exp OR incidence OR 'burden of disease'/exp OR 'burden of disease' OR (('burden'/exp OR burden) AND of AND ('disease'/exp OR disease)) OR 'disease burden'/exp OR 'disease burden' OR (('disease'/exp OR disease) AND ('burden'/exp OR burden)) OR 'burden of illness'/exp OR 'burden of illness' OR (('burden'/exp OR burden) AND of AND ('illness'/exp OR illness)) OR occurrence OR 'diagnosis'/exp OR diagnosis OR 'global burden of disease burden' OR (('global'/exp OR global) AND of AND ('disease'/exp OR disease) AND ('burden'/exp OR burden)) OR 'mortality'/exp OR ('mortality'/exp OR mortality OR 'death rate'/exp OR 'death rate' OR (('death'/exp OR death) AND rate) OR 'mortality rate'/exp OR 'mortality rate' OR (('mortality'/exp OR mortality) AND rate) OR 'death'/exp OR death OR 'survival'/exp OR survival OR 'fatality'/exp OR fatality OR 'laryngeal edema'/exp OR 'laryngeal edema' OR (laryngeal AND ('edema'/exp OR edema)) OR 'asphyxia'/exp OR asphyxia OR 'suffocation'/exp OR suffocation OR 'airway obstruction'/exp OR 'airway obstruction' OR (('airway'/exp OR airway) AND ('obstruction'/exp OR obstruction) OR 'healthcare resource utilization' OR (('healthcare'/exp OR healthcare) AND resource AND ('utilization'/exp OR utilization)) OR 'cost of illness'/exp OR 'cost of illness' OR (('cost'/exp OR cost) AND of AND ('illness'/exp OR illness)) OR 'cost'/exp OR cost OR costs OR 'productivity'/exp OR productivity OR economic OR 'economic burden'/exp OR 'economic burden' OR (economic AND ('burden'/exp OR burden)) OR 'healthcare costs' OR (('healthcare'/exp OR healthcare) AND costs) OR 'hospitalization'/exp OR hospitalization OR 'health resource utilization'/exp OR 'health resource utilization' OR (('health'/exp OR health) AND resource AND ('utilization'/exp OR utilization)) OR 'utilization'/exp OR utilization OR 'healthcare utilization'/exp OR 'healthcare utilization' OR (('healthcare'/exp OR healthcare) AND ('utilization'/exp OR utilization)) OR 'health service utilization'/exp OR 'health service utilization' OR (('health'/exp OR health) AND service AND ('utilization'/exp OR utilization)) OR 'direct cost'/exp OR 'direct cost' OR (direct AND ('cost'/exp OR cost)) OR 'indirect cost'/exp OR 'indirect cost' OR (indirect AND ('cost'/exp OR cost)) OR 'quality of life'/exp OR 'quality of life' OR (('quality'/exp OR quality) AND of AND ('life'/exp OR life)) OR qol OR hrqol OR 'health-related quality of life'/exp OR 'health-related quality of life' OR ('health related' AND ('quality'/exp OR quality) AND of AND ('life'/exp OR life)) OR 'health related quality of life'/exp OR 'health related quality of life' OR (('health'/exp OR health) AND related AND ('quality'/exp OR quality) AND of AND ('life'/exp OR life)) OR 'life quality'/exp OR 'life quality' OR (('life'/exp OR life) AND ('quality'/exp OR quality)) OR 'activities of daily living'/exp OR 'activities of daily living' OR (activities AND of AND daily AND ('living'/exp OR living)) OR 'patient satisfaction'/exp OR 'patient satisfaction' OR (('patient'/exp OR patient) AND ('satisfaction'/exp OR satisfaction)) OR 'caregiver burden'/exp OR 'caregiver burden' OR (('caregiver'/exp OR caregiver) AND ('burden'/exp OR burden)) OR 'impact of burden' OR (('impact'/exp OR impact) AND of AND ('burden'/exp OR burden)) OR 'quality adjusted life year'/exp OR 'quality adjusted life year' OR (('quality'/exp OR quality) AND adjusted AND ('life'/exp OR life) AND year))

WANGFANG

遗传性血管性水肿

CNKI

主题：遗传性血管性水肿
